# Draft Genome Sequence of the Probiotic Yeast *Saccharomyces cerevisiae* var. *boulardii* Strain ATCC MYA-796

**DOI:** 10.1128/genomeA.01345-14

**Published:** 2014-12-18

**Authors:** T. M. Batista, E. T. A. Marques, G. R. Franco, B. Douradinha

**Affiliations:** a Departamento de Bioquímica e Imunologia, Universidade Federal de Minas Gerais, Belo Horizonte, Brazil; b Fondazione RiMED, Palermo, Italy; c University of Pittsburgh, Center of Vaccine Research, Pittsburgh, Pennsylvania, USA

## Abstract

*Saccharomyces boulardii* is the only yeast approved as a probiotic for human consumption. Here, we report the draft genome sequence of the strain ATCC MYA-796, derived from the French Ultra Levure probiotic drug. The genome has a size of 11.6 Mb with 5,305 putative open reading frames predicted.

## GENOME ANNOUNCEMENT

Isolated by the French scientist Henri Boulard in 1920 during a cholera outbreak, the yeast *Saccharomyces cerevisiae* var. *boulardii* is still the only eukaryotic microorganism used as a probiotic in human health for the treatment of gastrointestinal disorders (1). This probiotic yeast is used worldwide and has been tested for clinical efficacy against several diseases, including traveler’s diarrhea, antibiotic-associated diarrhea (AAD), acute adult diarrhea, HIV-related diarrhea, *Helicobacter pylori* diseases, *Clostridium difficile* and *Salmonella typhi* infections, and Crohn’s disease, among others (2). Recently, a work was published describing the genome of an *S. boulardii* strain commercialized in India (3).

Here, we report the draft genome sequence of *S. cerevisiae* var. *boulardii* strain ATCC MYA-796, derived from the French Ultra Levure probiotic drug. The genomic DNA was sequenced using Illumina HiSeq by Axeq Technologies (http://www.axeq.com). A total of 48.3 million paired-end reads of 101 bp with an estimated 403× coverage were produced. Different k-mer values were tested to obtain the ideal value (k 61). The *de novo* assembly was performed using SOAPdenovo version 2.04 (4) with parameters -R -u -F -M 2. The contigs were scaffolded using SSPACE (5) and ordered by CONTIGuator (6) using the *S. cerevisiae* S288c genome as a reference; gaps were closed with GapCloser (4). The resulting assembly has 193 contigs (>400 bp) with a total length of 11,405,855 bp (the largest contig having a length of 459,679 bp), an *N*_50_ of 203 kb (in 19 contigs), and a G+C content of 38.1% and 100% of completeness, as estimated by mapping the orthologous genes (KOG databases) using CEGMA software (7). Genome annotation performed by MAKER2 (8) revealed 5,305 putative open reading frames (ORFs). An automatic annotation using the BLASTp algorithm (9) revealed 5,321 ORFs with significant similarity (*E*-value cutoff ≤10^−3^) to sequences deposited in the non-redundant (nr) protein database from NCBI. Using the tRNAscan-SE version 1.3 software (9), we found 273 tRNA genes scattered across the contigs.

The draft sequence of the yeast *S. cerevisiae* var. *boulardii* strain ATCC MYA-796 complements the genetic information of this probiotic yeast already deposited in GenBank.

### Nucleotide sequence accession numbers.

Data related to the whole-genome shotgun project of *S. cerevisiae* var. *boulardii* ATCC MYA-796 has been deposited at DDBJ/EMBL/GenBank under the accession number JRHY00000000. The version herein described is under accession number JRHY01000000.
